# Antimicrobial resistance in the Pacific Island countries and territories

**DOI:** 10.1136/bmjgh-2020-002418

**Published:** 2020-04-28

**Authors:** Michael J Loftus, Andrew J Stewardson, Ravi Naidu, Ben Coghlan, Adam WJ Jenney, Jonila Kepas, Evelyn Lavu, Alex B Munamua, Trisha N Peel, Vinita Sahai, Rosemary Tekoaua, Litia Tudravu, Julie Zinihite, Allen C Cheng, Eric Rafai, Anton Y Peleg

**Affiliations:** 1 Department of Infectious Diseases, The Alfred Hospital and Central Clinical School, Monash University, Melbourne, Victoria, Australia; 2 Colonial War Memorial Hospital, Suva, Fiji; 3 Health Security Program, Burnet Institute, Melbourne, Victoria, Australia; 4 College of Medicine, Nursing and Health Sciences, Fiji National University, Suva, Fiji; 5 Medical Standards Division, Government of Papua New Guinea National Department of Health, Port Moresby, National Capital District, Papua New Guinea; 6 Central Public Health Laboratory, Government of Papua New Guinea National Department of Health, Port Moresby, National Capital District, Papua New Guinea; 7 National Referral Hospital, Honiara, Solomon Islands; 8 Laboratory Services, Government of the Republic of Kiribati Ministry of Health and Medical Services, Tarawa, Kiribati; 9 National Pharmacy Division, Solomon Islands Ministry of Health and Medical Services, Honiara, Solomon Islands; 10 School of Public Health and Preventive Medicine, Monash University, Melbourne, Victoria, Australia; 11 Fiji Ministry of Health and Medical Services, Suva, Fiji; 12 Infection and Immunity Program, Monash Biomedicine Discovery Institute, Department of Microbiology, Monash University, Clayton, Victoria, Australia

**Keywords:** antimicrobial resistance, surveillance, antimicrobial use, antimicrobial stewardship, pacific island countries and territories

## Abstract

Antimicrobial resistance (AMR) is a critical global health threat with a disproportionate impact on low-income and middle-income countries (LMICs) due to their higher burden of infections, reduced laboratory surveillance infrastructure and fewer regulations governing antimicrobial use among humans or animals. While there have been increasing descriptions of AMR within many LMICs in WHO’s Western Pacific and South East Asian regions, there remains a paucity of data from Pacific Island countries and territories (PICTs). The PICTs represent 22 predominantly middle-income countries and territories with a combined population of 12 million people and 20 official languages, spread over hundreds of separate islands spanning an area corresponding to more than 15% of the earth’s surface. Our paper outlines the present state of the evidence regarding AMR in PICTs—discussing the present estimates of AMR and their accompanying limitations, important drivers of AMR, as well as outlining key priorities and potential solutions for tackling AMR in this region. Significant areas for action include developing National Action Plans, strengthening laboratory surveillance systems and educational activities targeted at both healthcare workers and the wider community. Ensuring adequate funding for AMR activities in PICTs is challenging given competing health and environmental priorities, in this context global or regional funding initiatives such as the Fleming Fund can play a key role.

**Summary box**

- There is a paucity of antimicrobial resistance (AMR) data from Pacific Island countries and territories (PICTs), especially for pathogens deemed ‘critical’ by WHO.
- Important potential drivers of AMR in PICTs include antimicrobial selection pressure in humans and animals, healthcare transmission of AMR pathogens, poor community understanding of AMR and increasing population movement and travel.
- In addressing AMR, a challenge for PICTs is trying to curtail antimicrobial *excess*, without jeopardising antimicrobial *access* for those who need them.
- Key priorities for PICTs responding to AMR should include developing National Action Plans, strengthening laboratory surveillance systems and educational activities targeted at both healthcare workers and the wider community.
- Providing funding for AMR can be difficult for PICTs in the setting of multiple competing health and environmental priorities; global or regional funding initiatives can play an important role.

## Introduction

The emergence of antimicrobial resistance (AMR) represents a growing health threat that could undo decades of medical progress. Previously innocuous infectious or routine surgical procedures, such as Caesarean sections, may become life-threatening events in a ‘postantibiotic era.’^1^ AMR is a truly global issue, resistant organisms do not respect national boundaries and are rapidly transmitted around the world. Additionally, while AMR is most often discussed in the context of human health, it is a broader, multifaceted problem with links to animal husbandry, agriculture, waste management and the environment.^2^ AMR poses a particularly strong threat to low-income and middle-income countries (LMICs), which frequently lack surveillance infrastructure to monitor for AMR, or regulations to govern the availability and use of antimicrobials for both humans and animals.^3^

**Figure 1:**
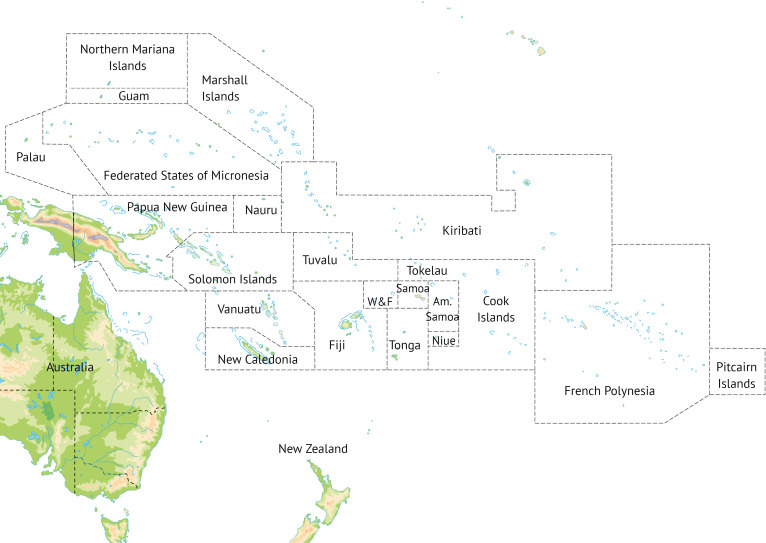
Map of Pacific Island countries and territories, Australia and New Zealand. Australia and New Zealand are not PICTs. Source: Shutterstock/frees. Reproduced with permission of Shutterstock. PICTs, Pacific Island countries and territories; W&F, Wallis and Futuna.

The Pacific Island countries and territories (PICTs) consist of 22 members of the Secretariat of the Pacific Community, with the omissions of Australia, New Zealand, USA and France (figure 1). All are members of the WHO Western Pacific Region. The populations of PICTs experience relative fragmentation and isolation: excluding Papua New Guinea (PNG), their combined landmass is only the size of Denmark, yet they consist of hundreds of islands extending over a region three times larger than Europe. Most PICTs share similar economic challenges as remote island economies far from major markets.^4^ Key industries include agriculture and aquaculture, tourism, forestry and, in select PICTs, mining.^5^ All countries in this region except Palau are defined by the World Bank as LMICs.^4^ The PICTs are also some of the most vulnerable in the world to the effects of natural disasters and climate change.^6^

The impact of AMR has been well described in other countries from the WHO Western Pacific Region^7 8^ or the neighbouring WHO South East Asian Region,^9^ however, there is a paucity of information about AMR within PICTs. This article seeks to provide a brief overview of the current state of knowledge regarding AMR in this region, and to highlight important challenges, key priorities and potential solutions to tackle AMR. We will not focus on AMR related to malaria, tuberculosis or HIV; these are distinct public health challenges and the focus of global disease-specific programmes.^10^

## Current human burden of AMR in PICTS

### Estimates of AMR prevalence

The existing knowledge of AMR in PICTs was summarised in a recent systematic scoping review identifying 65 relevant studies published since 1950.^11^ Reported rates of ‘critical’ Priority Pathogens^12^ varied widely between PICTs. For example, the proportion of *Escherichia coli* resistant to third-generation cephalosporins was 0% in Kiribati (n=72), 12% in Fiji (n=2895), 24% in (PNG, n=174) and 77% in Micronesia (n=158).^10^ Most of these estimates are well below the equivalent proportions reported in neighbouring AMR ‘hotspots’ such as India (20%–95%, n=10 247) or China (66%, n=113 892).^10^

To date, carbapenem resistance—one of the most challenging and concerning examples of AMR—appears to be uncommon. For example, less than 1% of 2175 *Klebsiella pneumoniae* surveillance isolates from a Fiji hospital were resistant to carbapenems.^10^ However, there have been multiple reports of outbreaks or individual cases of carbapenem-resistant infections in PICTs,^13–15^ raising the possibility of underdetection.

### Limitations of existing AMR estimates

Accurately defining the magnitude of the problem of AMR in PICTs using existing literature has many challenges (box 1). First, over two-thirds of the publications on AMR come from just three PICTs: Fiji, New Caledonia and PNG.^11^ Second, most studies are single centre, and often focus only on certain demographic groups (eg, children) or settings (eg, intensive care units), thus limiting the generalisability of results. Third, there are few contemporary publications, with the majority published prior to 2010.^11^ Fourth, due to limited resources, microbiological sampling may be reserved for the sickest patients or those failing antimicrobials—this can lead to an underestimate of overall infections but an overestimate of the proportion of AMR.^16^ Finally, the absence of resources for systematic and quality microbial surveillance is a further limitation. As in other LMICs, many smaller hospitals or clinics lack sufficient microbiology facilities or trained staff to perform antimicrobial susceptibility testing (AST), and those laboratories that are equipped for AST may face supply shortages or inadequate quality-assurance mechanisms.^17^ These factors can contribute to certain populations—especially rural or remote communities—being underrepresented in AMR surveillance.

Box 1 **Challenges when estimating prevalence of antimicrobial resistance (AMR) in Pacific Island countries and territories (PICTs)**

Limited volume of data

- Small number of published studies.
- Small number of tested isolates.
- Paucity of surveillance data especially notable for some ‘critical’ bug/drug combinations (eg, carbapenem-resistant *Acinetobacter baumannii*).

Limited generalisability of data

- Few AMR estimates published within last 5 years.
- Over-representation of certain PICTs (eg, Fiji, New Caledonia and Papua New Guinea) or subgroups (eg, paediatrics).
- Under-representation of populations with limited or absent *access* to antimicrobial susceptibility testing.

Limited quality of data

- Many PICT laboratories lack rigorous quality assurance processes.
- Many PICT laboratories face shortages of necessary equipment or trained staff.
- Subsequent potential for AMR to be missed or misclassified.

Long-term successful AMR surveillance in PICTs is possible, however, requires significant resources, infrastructure, capacity building and support. One example is the WHO Gonococcal Antimicrobial Surveillance Programme, active in certain PICTs for over 25 ye ars.^18^ In contrast, no PICTs have yet contributed data to the WHO Global Antimicrobial Resistance Surveillance System (GLASS).^17^

## Drivers of AMR dissemination in PICTS

**Figure 2:**
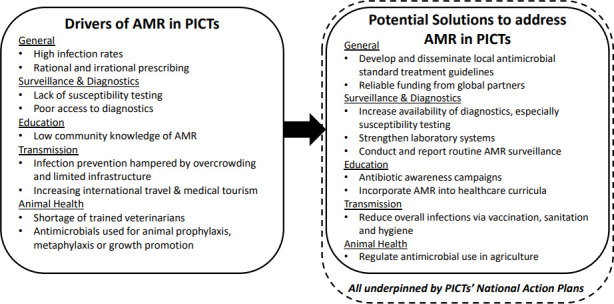
Drivers of and potential solutions for antimicrobial resistance (AMR) in Pacific Island countries and territories (PICTs).

From the global literature, we have extrapolated some of the main drivers that are likely to influence the development and spread of AMR in the Pacific. Under the headings below we have provided context and highlighted the existing state of knowledge in PICTs. A schematic representation of drivers of AMR and potential solutions is presented in figure 2.

### Antimicrobial selection pressure in humans

Antimicrobial use can exert selective pressure on micro-organisms, promoting the development and persistence of AMR isolates. Human antimicrobial consumption continues to rise globally, driven mostly by increases among LMICs.^19^ Accurately estimating consumption within LMICs can be challenging, as little or no prescribing data may exist, many antimicrobials are obtained without a prescription, and there may be parallel market sources.^20^ According to a recent WHO survey only two PICTs have regular antimicrobial monitoring and reporting systems in place.^21^ Furthermore, no PICTs contributed data to a recent global survey of antimicrobial consumption.^22^ The most comprehensive published estimate comes from Samoa and revealed a very high rate of antimicrobial consumption. In 2004, Samoa averaged 37.3 defined daily doses per 1000 inhabitants, higher than any European country at that time.^23^

### Antimicrobial selection pressure in animals

Use of antimicrobials in animals can also contribute to AMR. Animals may be given antimicrobials not only to treat existing infections, but also for prophylaxis, metaphylaxis (treating an entire group after a subset develop symptoms or disease) or growth promotion. There is little data on rates of antimicrobial use in animals in PICTs. Only five PICTs are member countries of the World Organisation for Animal Health (OIE), and the OIE’s annual report on regional antimicrobial use in animals provides aggregated data only, not country-level data.^24^ Just one country, Fiji, reports collecting detailed antimicrobial consumption data down to the farm level.^21^ There is a shortage of veterinarians within PICTs, compounded by a paucity of available diagnostic testing,^25^ which can both contribute to inappropriate antimicrobial use or selection of an inappropriate agent. Most PICTs lack government regulation of antimicrobial use in animals. There is also scarce data on AMR within food production systems. One New Caledonian study detected *Campylobacter* carriage among 97% of chicken neck-skins at the point of slaughter. Of concern 47% of isolates were resistant to ciprofloxacin, an important antimicrobial regularly used in humans.^26^

### Transmission of AMR pathogens

AMR acquisition can occur not only due to antimicrobial selective pressure, but also the direct transfer of multidrug-resistant organisms (MDROs) between humans, animals or the environment. This is a particular concern in healthcare settings, where high volumes of antimicrobials are used, infections are more likely to be due to MDROs and organisms can easily spread from patient to patient either directly or via healthcare workers and hospital equipment. Healthcare-associated infections are at least twice as common in LMICs than in high-income settings.^27^ In LMICs, infection prevention efforts face numerous challenges including overcrowding, low staffing, minimal training, a shortage of physical infrastructure (such as toilets or sinks), inadequate resources for consumables (such as gowns or alcohol-based handrub) and interrupted supply chains.^28^ Within PICTs there have been numerous reports of MDRO outbreaks, especially in intensive care units.^14 15 29^ Strict enforcement of infection control strategies has assisted in resolving the outbreak in each case.

### Community and prescriber understanding of AMR

Poor community knowledge about the purpose of antimicrobials, and the risks of AMR, can drive inappropriate demand for these products. Similarly, unnecessary prescribing of antimicrobials—for instance, for syndromes that are predominantly viral in aetiology—also contributes to excessive antimicrobial use. A survey of 112 Samoans living in New Zealand demonstrated common misconceptions around the role of antimicrobials: over 80% believed they could treat common colds, and very few (8%) were aware of the concept of AMR.^30^ Similarly, a community survey of 4995 Fijians showed that over half of recent antimicrobial courses were for upper respiratory tract infections, and less than a third of respondents knew what AMR was. Antimicrobial knowledge strongly correlated with level of education.^31^ High rates of unnecessary prescribing was also confirmed in a recent paediatric outpatient study in PNG: 82% of children presenting with a common cold were given antimicrobials.^32^

### Human movement and travel

Traditionally, PICTs experienced low volumes of airline traffic due to their geographical dispersion, small populations and high costs of fuel and parts. However, the recent emergence of low-cost carriers has led to greater capacity on existing airline routes and the introduction of new routes.^33^ This has resulted in increased opportunities for PICT citizens to travel to surrounding regions, as well as annual inbound tourism growth rates above 10% for many PICTs^33^—with increasing visitors from non-traditional markets such as China and Russia.^34^ These greater population movements provide economic opportunities for PICTs, but do increase the risk of introducing AMR.

Another potential transmission source of AMR for PICTs is medical tourism.^35^ Certain procedures—such as renal transplantation—are not available in PICTs, necessitating either self-funded or government-supported overseas travel to access care. A common destination is India,^36^ a country with high reported AMR prevalence^37^ and an associated risk that patients return colonised or infected with MDROs. Of note, most PICTs do not screen patients returning from overseas healthcare for MDROs, so this potentially significant AMR risk cannot be quantified or mitigated.

## Key priorities and potential solutions for PICTS

AMR is an important global health threat to which PICTs are particularly vulnerable. Globally, the danger of AMR has been recognised and efforts made to mobilise a response. The WHO’s ‘Global Action Plan on AMR’ is the internationally agreed framework for action.^1^ It consists of five key objectives, which include: (1) improving AMR awareness; (2) strengthening knowledge by surveillance and research; (3) reducing infection by sanitation, hygiene and infection prevention; (4) optimising antimicrobial use among humans and animals; and (5) developing the economic case for sustainable investment and increasing investment in new medicines, diagnostics, vaccines and other interventions. Viewing the PICTs through the prism of the WHO’s Global Action Plan, we have highlighted below what we believe should be priorities for this region in tackling AMR. A summary of how PICTs has responded to key AMR issues to date is presented in table 1.

**Table 1:** Summary of key issues relating to AMR in PICTs

Issue related to AMR in PICTs	Strengths/achievements	Weaknesses/limitations
National Action Plans (NAPs) for AMR	NAPs approved by government in Fiji and Cook Islands	Many PICTs remain without well-developed NAPs
Laboratory surveillance capacity	Long-standing *Neisseria gonorrhoeae* surveillance in select PICTs	Antimicrobial susceptibility testing typically only available in large centres WHO GLASS report contains no data from PICTs
Surveillance of antimicrobial consumption (human)	Extensive use of mSupply in Pacific holds potential for real-time monitoring of consumption	WHO report on Surveillance of Antimicrobial Consumption contains no data from PICTs
Standard treatment guidelines (STGs)	Antimicrobial STGs available in at least eight PICTs Fiji and Solomon Islands STGs freely available via smartphone app	Presence of STGs doesn’t guarantee adherence—adherence to STGs in Solomon Islands has improved but remains at 44%^50^
Community education	Most PICTs participate in ‘Antibiotic Awareness Week’ each November	No assessment of effectiveness of these campaigns
Surveillance of antimicrobial consumption (animal)	Fiji alone reports collecting data on antimicrobial use in animals down to farm and species level	Paucity of reported data
Animal health	Presence of Food and Agriculture Organisation of the United Nations, with subregional office in Samoa since 1996	Lack of government regulation restricting antimicrobial use in animals

AMR, antimicrobial resistance; GLASS, Global Antimicrobial Resistance Surveillance System; PICTs, Pacific Island countries and territories.

### Developing National Action Plans on AMR

The Global Action Plan calls on countries to develop National Action Plans (NAPs) that are aligned with the global strategy but take into account local resources, national priorities and local governance arrangements.^1^ To date, only two PICTs (Fiji and the Cook Islands) have had NAPs approved by government. A few other PICTs have NAPs under development awaiting government approval.^21^ While the presence of a NAP does not mean it is implemented—and similarly the absence of a NAP does not prevent important action on AMR—NAPs help formalise and facilitate important cross-sectoral cooperation between different government departments such as Health, Agriculture and Environment. These collaborations can have benefits beyond AMR, for instance, improved responses to zoonoses such as leptospirosis.

### Improving laboratory infrastructure and surveillance capacity

Regular and reliable surveillance should be the cornerstone of any AMR response. It provides an accurate understanding of AMR prevalence and changes over time, permitting the development of targeted AMR policies informed by local data. However, as highlighted earlier, AMR surveillance in PICTs can be variable, hindered by a lack of trained staff or consistent access to the necessary equipment and materials. Currently data are often recorded in hard copy, and cannot rapidly be linked to a centralised system or other facilities. PICTs should aim to develop their national laboratory infrastructure so that AST can be reliably performed on WHO Priority Pathogens^12^ among human isolates, and these data contributed to global reporting systems such as GLASS.^17^ Laboratory surveillance capacity for animal health in PICTs is even further underdeveloped, efforts should be made to promote and support increased testing for AMR within the animal sector.

### Improving antimicrobial stewardship and community awareness

Correct selection of antimicrobials by healthcare providers is also important to reduce AMR. Such stewardship is assisted by targeted education of healthcare workers, and developing standard treatment guidelines.^38^ Presently, antimicrobial guidelines exist in at least eight PICTs including Fiji, Solomon Islands, Cook Islands, Samoa, Vanuatu, Tonga, Marshall Islands and Kiribati, with further guidelines under development. Other PICTs such as PNG provide some antimicrobial recommendations within the context of broader standard treatment guidelines. The Australian Therapeutic Guidelines Foundation has created mobile apps for both Fiji and Solomon Islands, making their antimicrobial guidelines freely available and easily accessible to any smartphone user.

Antimicrobial prescribing can also be optimised by rapidly differentiating between bacterial infections and other febrile illnesses. Studies from LMICs in South East Asia have demonstrated modest reductions in antimicrobial use with point-of-care testing for C-reactive protein^39^ or influenza.^40^ Financial costs and staff training are some of the barriers to widespread use of such diagnostics-based stewardship in PICTs.

Awareness campaigns can help reduce unnecessary demand for antimicrobials, especially in the outpatient setting.^41^ Many PICTs have developed such campaigns, with events usually centred around World Antibiotic Awareness Week each November. Campaigns should be adapted to the local context,^42^ an example is Samoa’s 2016 ‘Fa’aaoga with love’ campaign which translates to ‘use (antibiotics) with love’.^43^ The effectiveness of antimicrobial awareness campaigns in PICTs has not been formally evaluated. Priorities should include sociological research to determine community motivations for antibiotic use, followed by assessment and subsequent tailoring of campaign messaging.

Lastly, when considering stewardship initiatives in PICTs, it is essential to recognise that more deaths currently occur in LMICs due to inadequate access to antimicrobials, rather than AMR.^44^ Therefore, any efforts to curtail antimicrobial *excess* should not concurrently deny antimicrobial *access* to those patients who require them.

### Securing funding for AMR initiatives among competing budget priorities

Allocating adequate funding towards AMR in PICTs is challenging. Competing national budget priorities—for instance, the rapid increase in non-communicable diseases,^45^ or threats such as climate change—may overshadow the risk of AMR. However, many of the financial expenses to address AMR, for example, improving laboratory IT or surveillance systems, would have additional benefits for the health system beyond AMR.

Globally, there has been a significant increase in funding for AMR research and initiatives in LMICs, most prominently through the Fleming Fund.^46^ However, PNG is currently the sole PICT among the Fund’s partner countries. Foreign aid remains an important source of financial support for PICTs. Donors are increasingly targeting funding at projects addressing outbreaks and other regional health threats such as AMR, for instance, through the Australian Government’s Health Security Initiative for the Indo-Pacific region.^47^

### Performing novel, real-time, detailed surveillance of antimicrobial use

The small size and relative isolation of PICTs can be advantageous when monitoring antimicrobial consumption: there is little parallel importation of medicines, and national coverage of dispensaries is easier to achieve. Presently, many PICTs monitor their stockpiles and movements of medicines using software such as mSupply,^48^ which is updated in real time by dispensing staff at individual clinics. Tupaia is a project that collates data from mSupply and other sources to present important healthcare data to both consumers and policy-makers, presently active in six PICTs.^49^

There is scope for this existing digital data to be analysed and presented as a proxy for antimicrobial consumption: providing accurate, real-time data down to the level of individual regions or clinics. In addition to the ability to contribute data to WHO’s Global Programme on Surveillance of Antimicrobial Consumption^22^; there are multiple other benefits from such a surveillance system. For example, changes in long-standing prescribing practices could be rapidly identified and investigated for the possibility of a disease outbreak. Alternatively, clinics or regions with prescribing patterns that fell outside of normal variation could be audited and provided with targeted education if required.

## Conclusions

Rates of both AMR and antimicrobial consumption are rising globally, especially within LMICs. Such increases are likely being replicated within PICTs, which face similar challenges including high rates of infectious diseases, low community knowledge around antimicrobials and AMR, and limited laboratory surveillance capacity. However, precise estimates of AMR prevalence within humans, and especially within animals, are hindered by a scarcity of data.

WHO has taken a leading role in coordinating a global response to AMR, most notably through its Global Action Plan. Within PICTs, we suggest that addressing AMR should initially focus on: (1) optimising governance and cross-sectoral collaboration through the establishment of NAPs, (2) optimising surveillance through strengthening laboratory capacity and (3) optimising antimicrobial awareness through community education activities and provision of standard treatment guidelines for clinicians. Additionally, we believe PICTs have the potential to rapidly develop advanced surveillance systems for antimicrobial consumption in humans by building on existing IT infrastructure and improving laboratory systems. While providing reliable funding for AMR activities may be challenging for PICTs in the setting of multiple competing health and environmental priorities, global or regional funding initiatives can play an important role. Technical and financial support for sustainable collaborations and international partnerships with organisations with established AMR surveillance systems can help reduce the barriers faced by PICTs.
